# Highly Stable Nonhydroxyl Antisolvent Polymer Dielectric: A New Strategy towards High-Performance Low-Temperature Solution-Processed Ultraflexible Organic Transistors for Skin-Inspired Electronics

**DOI:** 10.34133/2021/9897353

**Published:** 2021-12-08

**Authors:** Mingxin Zhang, Cong Zhang, Yahan Yang, Hang Ren, Junmo Zhang, Xiaoli Zhao, Yanhong Tong, Qingxin Tang, Yichun Liu

**Affiliations:** Centre for Advanced Optoelectronic Functional Materials Research and Key Laboratory of UV-Emitting Materials and Technology, Ministry of Education, Northeast Normal University, Changchun 130024, China

## Abstract

Scarcity of the antisolvent polymer dielectrics and their poor stability have significantly prevented solution-processed ultraflexible organic transistors from low-temperature, large-scale production for applications in low-cost skin-inspired electronics. Here, we present a novel low-temperature solution-processed PEI-EP polymer dielectric with dramatically enhanced thermal stability, humidity stability, and frequency stability compared with the conventional PVA/c-PVA and c-PVP dielectrics, by incorporating polyethyleneimine PEI as crosslinking sites in nonhydroxyl epoxy EP. The PEI-EP dielectric requires a very low process temperature as low as 70°C and simultaneously possesses the high initial decomposition temperature (340°C) and glass transition temperature (230°C), humidity-resistant dielectric properties, and frequency-independent capacitance. Integrated into the solution-processed C8-BTBT thin-film transistors, the PEI-EP dielectric enables the device stable operation in air within 2 months and in high-humidity environment from 20 to 100% without significant performance degradation. The PEI-EP dielectric transistor array also presents weak hysteresis transfer characteristics, excellent electrical performance with 100% operation rate, high mobility up to 7.98 cm^2^ V^−1^ s^−1^ (1 Hz) and average mobility as high as 5.3 cm^2^ V^−1^ s^−1^ (1 Hz), excellent flexibility with the normal operation at the bending radius down to 0.003 mm, and foldable and crumpling-resistant capability. These results reveal the great potential of PEI-EP polymer as dielectric of low-temperature solution-processed ultraflexible organic transistors and open a new strategy for the development and applications of next-generation low-cost skin electronics.

## 1. Introduction

Low-temperature solution-processed flexible organic field-effect transistors (OFETs) are expected to open up application opportunities in new-generation low-cost skin-inspired electronics [1–9]. To realize such OFETs, the dielectric must be antisolvent to resist dissolution or swelling in the deposition process of the organic semiconductor layer. As a result, only the limited antisolvent polymers, mainly including Polyimide (PI), polyethylene terephthalate (PET), poly (vinyl alcohol) (PVA/c-PVA), and poly (4-vinylphenol) (c-PVP), have been shown as dielectrics of low-temperature solution-processed flexible OFETs. Compared with PI and PET, c-PVP and PVA/c-PVA as dielectric layers require the lower precursor conversion temperature only at 70-100°C [10, 11] and hence have been more popular in solution-processed flexible and conformal OFETs. However, one of the major remaining issues is instability of dielectrics. The residual hydroxyl groups in PVA/c-PVA and c-PVP dielectrics have been found to absorb water in air, act as trap sites, and cause slow polarization, resulting in significant performance deterioration of transistors [12]. The poor operational stability, presented by fluctuation, shift, and hysteresis in transfer curves, has been extensively observed in c-PVP and PVA/c-PVA dielectric OFETs [13, 14]. The sensitivity of PVA/c-PVA and c-PVP to moisture also drastically alters the dielectric properties leading to high leakage current density and low carrier mobilities in humidity environment [15].

On the other hand, the frequency-dependent capacitance of the polymer dielectrics has been observed in many dielectrics such as TPU, PVDF-HFP, ion gel, and polyelectrolyte [16, 17]. The slow polarization velocity of these polymer dielectrics makes much fewer carriers accumulated at the dielectric/semiconductor interface of transistors at high frequency, i.e., lower capacitance at high frequency. The dielectric capacitance is typically measured at high frequency (10^3^ Hz) while transistor measurements are usually carried out at quasistatic low-frequency conditions (0.1-1 Hz) [18]. As a result, the standard Metal-Oxide-Semiconductor Field-Effect Transistor (MOSFET) model calculation procedure in literatures for charge carrier mobility of OFETs using a high-frequency capacitance value causes the overestimation of mobility even by orders of magnitude [19]. At the same time, circuit design requires transistor to operate stably over a wide frequency range. Therefore, the frequency-stable dielectric capacitance value has been actively pursed for fundamental studies and practical applications.

Here, for the first time, a novel nonhydroxyl PEI-EP antisolvent polymer dielectric is synthesized at 70°C for low-temperature solution-processed ultraflexible OFETs. To show its outstanding advantages, PEI-EP and the corresponding field-effect performance are compared with that of the conventional c-PVP and PVA/c-PVA dielectrics. The PEI-EP dielectric presents significantly improved thermal stability, humidity stability, and frequency stability. The resulting ultraflexible C8-BTBT transistor array also shows dramatically improved electrical performance with the weak hysteresis and stable operation in air within 2 months and in high-humidity environment from 20 to 100%. These combined with 100% operation rate, high mobility up to 7.98 cm^2^ V^−1^ s^−1^, and average mobility as high as 5.3 cm^2^ V^−1^ s^−1^ present a promising potential of PEI-EP as the dielectric material for solution-processed ultraflexible organic electronics.

## 2. Results and Discussion

### 2.1. Synthesis of Ultraflexible PEI-EP Dielectric

**Figure 1:**
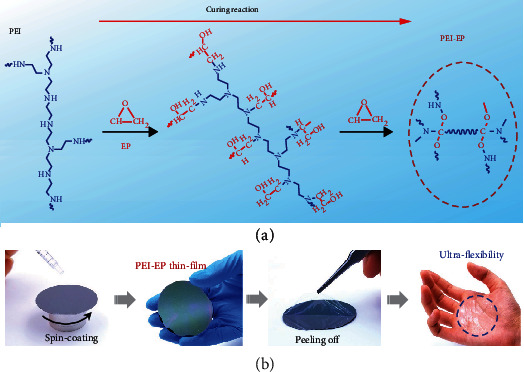
(a) A schematic illustration of the synthesis of PEI-EP film through the curing reaction. (b) Fabrication of solution-processed ultraflexible PEI-EP polymer thin film.

Figure 1(a) illustrates the synthesis route and curing reaction of PEI-EP, and Figure 1(b) shows the fabrication process of ultraflexible PEI-EP thin film by spin-coating the mixing precursor solution of PEI and EP in chloroform followed by a low-temperature annealing process at 70°C for 2 h. At 70°C, the epoxy groups in EP are opened by the amine groups in PEI, which results in the generation of the hydroxyl groups and more secondary/tertiary amine groups. The newly formed hydroxyl groups further react with the epoxy groups in EP. As a result, a crosslinking network with both amine groups and ether linkages is obtained [20, 21]. The FTIR spectrum of PEI-EP in Figure S1 shows the absorbing peak of C-O-C at 1750 cm^−1^, indicating that PEI-EP is successfully synthesized at low temperature of 70°C [22]. More importantly, the absorption peak of the hydroxyl group at 3400 cm^−1^ does not appear in the FTIR spectrum, confirming the hydroxyl-free characteristic of PEI-EP.

To investigate the flexibility of PEI-EP, as shown in Figure 1(b), the PEI-EP thin film was spin-coated on the octadecyltrichlorosilane (OTS) modified Si wafer at room temperature, followed by peeling off and then attaching on a human hand. The PEI-EP thin film presents good conformability on the human hand simultaneously with the high transparency of over 98% at visible light, as shown in Figure 1(b) and Figure S2, showing the outstanding application potential in new-generation invisible wearable electronics.

### 2.2. Solvent Resistance, Thermal Stability, and Humidity Resistance of PEI-EP

**Figure 2:**
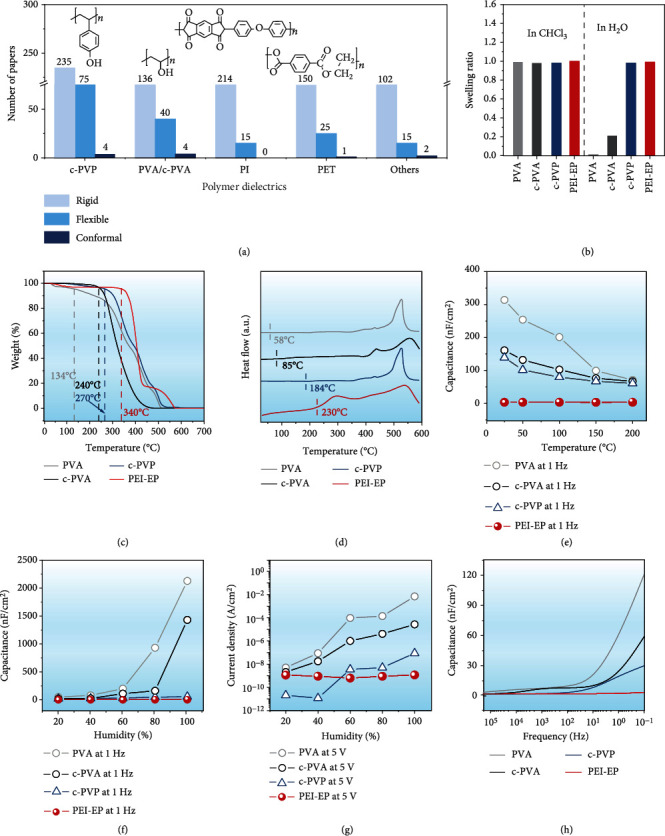
Solvent resistance, thermal stability, and humidity resistance properties of PEI-EP dielectric compared with commercial polymer dielectrics including PVA/c-PVA and c-PVP. (a) Statistics of published literatures based on solution-processed polymer dielectric organic transistors. (b) Swelling ratios in CHCl_3_ and H_2_O. (c) TG curves from 0 to 700°C. (d) DSC curves from 0 to 600°C. (e) Capacitance from 25 to 200°C at 1 Hz. (f) Capacitance from 20 to 100% humidity at 1 Hz. (g) Leakage current density from 20 to 100% humidity at 5 V. (h) Capacitance from 200 kHz to 0.1 Hz.

More interesting, our results show that PEI-EP is a highly stable insulating material that can resist organic solvents, temperature, and humidity. These outstanding advantages enable PEI-EP promising dielectric candidate for solution-processed flexible OFETs. As mentioned above, the available dielectric materials for solution-processed flexible OFETs are scarce.  Figure 2(a) illustrates the number of literatures for the polymer dielectrics reported in solution-processed organic transistors in the past 10 years. Among them, c-PVP, PVA/c-PVA, PI, and PET are the most frequently used flexible polymer dielectrics. PI and PET face the challenging of high process temperature at 275-300°C, which is much higher than the deposition temperature of organic semiconductors (<120°C) and not favorable for low-cost production [23]. In contrast, PVA/c-PVA and c-PVP require a low process temperature at 70-100°C and have been more extensively applied into solution-processed flexible and conformal organic transistors, as shown in Figure 2(a). Therefore, our PEI-EP dielectric with the low-temperature process (70°C) presents the outstanding advantage for efficient low-cost industrialization production.

To show the promising potential of the PEI-EP polymer as dielectric of solution-processed flexible OFETs, here, we investigate the stability of the PEI-EP dielectric and compare its characteristics with the conventional PVA/c-PVA and c-PVP dielectrics in Figures 2(b)–2(h).  Figure 2(b) shows the antisolvent capability of PEI-EP by swelling ratio (volume ratio of material before and after immersion in solvent). If the swelling ratio of the material in solvent, for example, the thermosetting polymers PDMS and vulcanized rubber in chloroform, is over 1, the solvent molecules can enter into the dielectric materials resulting in the volume increase (i.e., swelling) and hence the failure in solution deposition of organic semiconductor thin films [24]. Here, the swelling ratio of all of dielectrics after immersing in chloroform is ~1, showing their strong resistance to chloroform. To further confirm excellent resistance of PEI-EP to more solvents, the PEI-EP films were also immersed into heptane and acetone for over 12 h; the swelling ratio and their Fourier transform infrared (FTIR) spectra are compared with that of the pristine PEI-EP film (Figure S4). The swelling ratios are still ~1, and the functional groups at 2916 cm^−1^ (C−H), 1727 cm^−1^ (C-O), and 1750 cm^−1^ (C-O-C) appear for all films, confirming that the PEI-EP dielectric remains with excellent stability with the unchanged molecular structures in different organic solvents. In Figure 2(b), the swelling ratios of PVA and c-PVA in H_2_O are ~0 and 0.2, respectively, showing the water-soluble property of PVA/c-PVA. In contrast, the swelling ratios of PEI-EP and c-PVP are ~1, indicating the good volume stability of PEI-EP and c-PVP in water.

When the organic flexible electronic devices are experiencing the high-temperature fabrication or operation process, the low thermal stability would make the devices vulnerable to damage [25]. Thermogravimetric Analysis (TG) in Figure 2(c) shows that the initial decomposition temperature (*T*_5%_, defined as the temperature at which 5% of weight loss occurs) of PVA, c-PVA, c-PVP, and PEI-EP is 134, 240, 270, and 340°C. Obviously, PEI-EP has much higher *T*_5%_ than PVA/c-PVA and c-PVP. Figure 2(d) shows obviously higher glass transition temperature for PEI-EP (230°C) than PVA/c-PVA and c-PVP (58/85 and 184°C, respectively), which combined with TG results confirms the obviously better thermal stability of PEI-EP. Further, we also investigated the thermal stability of capacitance at 1 Hz for four dielectrics in Figure 2(e). The dielectric capacitance of OFETs affects the number of the induced charges at the semiconductor/dielectric interface and therefore affects the carrier transport and operation stability of devices. The capacitance of PVA/c-PVA and c-PVP is sharply decreased from 25 to 200°C. In contrast, the capacitance of PEI-EP almost remains unchanged with the temperature, presenting the stable dielectric properties.

Humidity is another key factor for dielectrics that directly affects the stability of OFETs. Here, the capacitance and leakage current density of PVA/c-PVA, c-PVP, and PEI-EP were measured from 20 to 100% humidity based on the metal-insulator-metal (MIM) device configuration, as shown in Figures 2(f) and 2(g). The capacitance of PVA/c-PVA sharply increases by over three orders of magnitude with the increased humidity. c-PVP presents the capacitance increased by 150% while PEI-EP shows only 25% change of the capacitance when the humidity changes from 20 to 100%. Figure 2(g) shows that the leakage current density of PVA/c-PVA and c-PVP dramatically increases by over 4 orders of magnitude when the humidity changes from 20% to 100%. In contrast, PEI-EP always remains the stable insulator property with the low leakage current density of 10^−9^ A/cm^2^ from 20% to 100% humidity. From Figure 2, it can be clearly seen that c-PVA presents the improved thermal stability and humidity stability compared with PVA, originating from the decreased number of hydroxyl groups by the crosslinking process [26]. However, the incomplete crosslinking with the residual hydroxyl groups still adsorbs water molecules [15]. With the increased humidity, more H_2_O molecules penetrate into the PVA/c-PVA and c-PVP films, resulting in the increased capacitance and leakage current density as the formation of the electric double layer (EDL) [27]. The capacitance change affects the stable operation of OFETs in circuits. The leakage current density affects the off-state current and on-off ratio of OFETs and weakens the modulation effect of the gate electric field. The high leakage current density even possibly breaks down the dielectric layer resulting in failure in device operation [28]. In contrast, the hydrophobic interface of hydroxyl-free PEI-EP (Figure S3) and its strong covalent bonds can effectively resist to water molecules [21]. Therefore, the PEI-EP always remains with good humidity stability of capacitance and good insulator property, as shown in Figures 2(f) and 2(g).

According to the standard MOSFET model, the calculated mobility value is inversely proportional to the measured capacitance. The changed capacitance of the dielectric with frequency generally causes overestimation of field-effect mobility in previous reports and failure in circuit design [19]. Figure 2(h) compares the frequency stability of capacitance of the four dielectrics. The dramatic capacitance difference between at high frequency (10^5^-10^2^ Hz) and at low frequency (100 to 0.1 Hz) can be observed in PVA/c-PVA and c-PVP dielectrics. In contrast, the capacitance-frequency (*C*‐*F*) plot of PEI-EP dielectric is relatively flatter, indicating the promising potential of PEI-EP as dielectric material of flexible organic electronics [28].

### 2.3. Solution-Processed C8-BTBT Flexible Transistor Array Based on PEI-EP Dielectric

**Figure 3:**
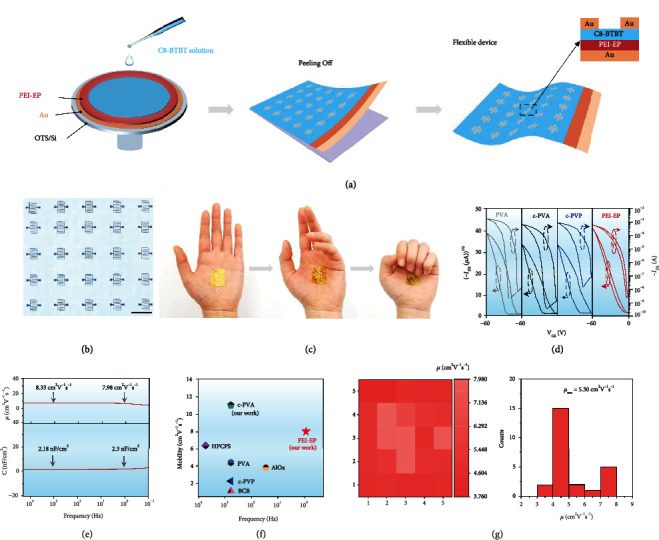
Ultraflexible C8-BTBT OFET array based on PEI-EP dielectric. (a) Fabrication schematic of ultraflexible PEI-EP dielectric OFET array. (b) Magnified transmission optical microscopy image of the 5 × 5 OFET array. (c) The ultraflexible PEI-EP dielectric transistor array adhered onto the human palm. (d) Double sweep transfer curves of PEI-EP dielectric compared with PVA/c-PVA and c-PVP dielectric transistors at 20% humidity. (e) Calculated mobility according to the capacitance measured from 200 kHz to 0.1 Hz. (f) The statistical results of the reported mobilities of C8-BTBT OFET measured capacitance at a frequency. (g) Color mappings and histogram distributions of mobilities of the transistor array extracted with the capacitance at 1 Hz.

In practical applications, the large-area production via solution-processed methods, with a high yield ratio of device fabrication, good device performance uniformity, and high performance, is more desirable [11]. To show the advantages of PEI-EP as dielectric for solution-processed flexible transistors, a 5 × 5 flexible OFET array was fabricated by the spin-coating method, as shown in Figure 3(a). It is worth noting that PEI-EP is first reported as polymer dielectric in organic thin-film transistors. C8-BTBT is used as the active layer because of its highly ordered molecular packing and efficient charge transport [29]. Firstly, 50 nm Au was deposited on OTS modified Si wafer by vacuum deposition. Next, the mixed solution of PEI and EP was spin-coated on Au and annealed at 70°C oven for 2 h to promote the crosslinking reaction for the formation of the PEI-EP dielectric layer. The surface roughness of PEI-EP is only 0.41 nm as shown in Figure S5, which is beneficial for increasing the carrier mobility [30]. After that, 5 mg/mL C8-BTBT solution was spin-coated on the PEI-EP dielectric layer as the active layer. It can be observed that the uniform C8-BTBT semiconductor layer can be directly formed on the PEI-EP dielectric layer by the solution process. Finally, 30 nm Au was deposited through a shadow mask as the source and drain electrodes, respectively. Finally, the whole device could be easily peeled off from the OTS/Si wafer. The resulting thickness of the OFET array is only 890 nm with the extremely ultralight weight at 0.2 mg/cm^2^ (Figures S6 and S7).

To obtain the optimized conditions, the concentration of C8-BTBT solution and spinning speed were, respectively, investigated by AFM images of the formed C8-BTBT thin films in Figures S8 and S9. The concentration was located at 3, 5, 8, and 10 mg/mL, and the spinning speed changed from 1000 to 8000 rpm. The C8-BTBT thin film at 5 mg/mL presents the lowest root-mean-square (RMS) value, which is beneficial to improve device performance [31]. With the increased spinning speeds and larger grain size, more continuous and more uniform C8-BTBT films with fewer crystal boundaries and lower RMS values are obtained. At the spinning speed of 6000 rpm, the lowest RMS value of C8-BTBT thin films is only 6.43 nm. Therefore, the ultraflexible C8-BTBT transistor array was prepared at the concentration of 5 mg/mL and the spinning speed of 6000 rpm. Figure 3(b) gives a typical transmission optical microscopy image of the PEI-EP dielectric C8-BTBT transistor array.

As shown in Figure 3(c), the PEI-EP dielectric ultraflexible transistor array can be seamlessly adhered onto a human palm and can remain with good attachment to the skin with the movement of the human palm, presenting huge application potential in electronic skin. To show the advantage of PEI-EP as the dielectric of OFETs in the improved stability, Figure 3(d) presents the double sweep transfer curves of PEI-EP dielectric C8-BTBT thin-film FETs at humidity of 20%. For comparison, the corresponding transfer curves of PVA/c-PVA and c-PVP dielectric devices are also shown in Figure 3(d). Obviously, large hysteresis during cyclic sweep can be observed in PVA/c-PVA and c-PVP dielectric OFETs, which is in good agreement with the previous reports [32, 33]. With the change of the electric field during sweeping, the polar hydroxy groups can make the carriers injected from the gate and trapped in dielectrics, thereby affecting the channel current and leading to a mismatch of forward and reverse gate sweeps in *I*_D_ − *V*_G_ curves [11, 18, 30]. In contrast, PEI-EP dielectric OFETs represent obviously improved electrical stability with the negligible hysteresis as a result of nonhydroxyl interface. Besides, the PEI-EP dielectric shows low gate leakage current compared with commercial PVA/c-PVA and c-PVP as shown in Figure S11.

Further, it is very critical to choose the proper measured capacitance at a frequency [32]. Even though researchers have recognized this problem, there has been no selection criteria for frequency to calculate mobility. As the electrical properties of OTFTs are typically acquired by quasistatic approaches, the capacitance values at the lowest frequency limit are most likely suitable for analyzing the field-effect mobility. However, limited by the tradition LCR meter, the lowest frequency is only 20 Hz, which is much higher than the quasistatic frequency, resulting in the unreliable mobility [34]. Zhenan Bao's group first reported the method of time constant of a resistor-capacitor (RC) circuit to measure the quasistatic capacitance. The results show large capacitance values in certain elastomers coming from the EDL formation as the mobile ions in the elastomers. In this scenario, the regular LCR meters cannot measure the low frequency to measure the capacitance of the ion polarization and transistor characteristics, resulting in the overestimation of the mobility by orders of magnitude [18]. Wei Huang's group also reported similar results. According to previous studies, somepolar groups such as hydrogen ions contribute to slow polarization of the dielectric, resulting in much higher capacitance at low frequencies versus the typical frequencies (≥10^3^ Hz) used to measure the dielectric capacitance. Thus, since TFT measurements are usually carried out at quasistatic conditions (<10 Hz), using the high-frequency capacitance value to extract the mobility significantly overestimates the field-effect mobility. In this scenario, it is necessary to calculate the mobility extracted from the quasistatic (<10 Hz) capacitance.

Figure 3(e) shows the calculated mobilities according to the capacitances measured at a wide frequency from 200 kHz to 0.1 Hz. For PEI-EP dielectric, the stable capacitance with frequency weakens the fluctuation of the calculated mobility. When the frequency decreases from 1000 to 1 Hz, the capacitance of PEI-EP increases weakly from 2.18 to 2.3 nF/cm^2^, resulting in the weak changes of the calculated mobility from 8.33 to 7.98 cm^2^ V^−1^ s^−1^. The high and stable mobility of the PEI-EP dielectric C8-BTBT thin-film transistors show the promising potential of the PEI-EP as dielectric for solution-processed flexible organic integrated circuits. For C8-BTBT thin-film OFETs, most of the reported literatures did not give the measured frequency, and only a few groups have addressed the measured capacitance values, respectively, at 10000, 1000, and 20 Hz and have given the calculated mobility values at 6.36, 1.13/2.25/4.36/11, and 3.8 cm^2^ V^−1^ s^−1^, as shown in Figure 3(f). For example, our previous work has shown the mobility of c-PVA dielectric C8-BTBT thin-film FETs as high as 11 cm^2^ V^−1^ s^−1^, which is extracted from the capacitance at 1000 Hz and actually corresponds to the mobility value only at 4.28 cm^2^ V^−1^ s^−1^ with the capacitance at 1 Hz. In Figures 3(e) and 3(f), the high calculated mobility extracted with the capacitance at the low frequency (7.98 cm^2^ V^−1^ s^−1^ at 1 Hz) presents the outstanding advantage of the PEI-EP as dielectric. At the same time, considering a regular Impedance analyzer failing in measurements at low frequency, the stable capacitance value with frequency in our PEI-EP dielectric is also very favorable to achieve the reliable mobility value [18].

To further show the high performance of our PEI-EP dielectric OFET array, the device performance uniformity is presented by the distribution of the field-effect mobility, and the corresponding statistical results are shown in Figure 3(g) (extracted with the capacitance at 1 Hz). The OFET array shows a good yield ratio as high as 100%, and the average saturation mobility is as high as 5.30 cm^2^ V^−1^ s^−1^.

### 2.4. Stability of PEI-EP Dielectric Organic Transistors

**Figure 4:**
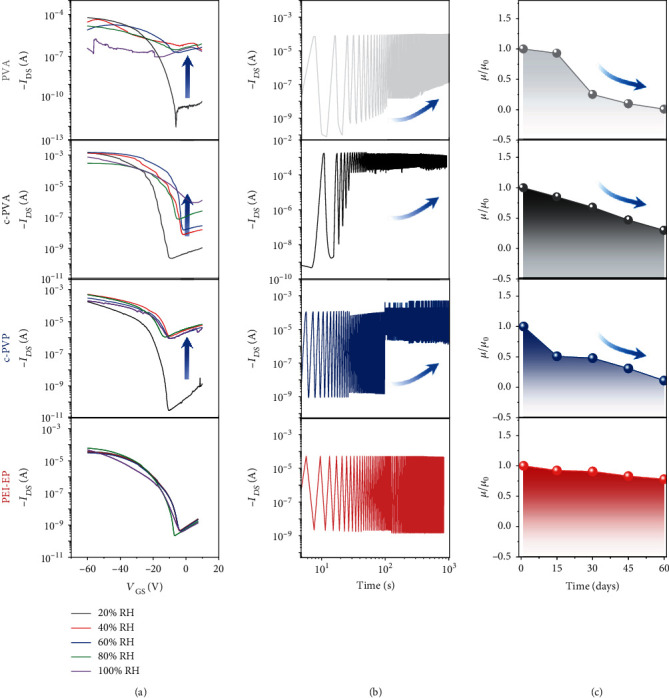
Stability of PEI-EP dielectric OFET compared with commercial PVA/c-PVA and c-PVP polymer dielectrics. (a) Transfer curves at the humidity from 20 to 100%. (b) Cycle stability of the transistors with continuous switch between on and off states for 1000 s at 20% humidity. (c) Time stability of the device mobility in 60 days.

Here, the dielectric effect can be directly observed by the electrical performance of OFETs. For PVA/c-PVA and c-PVP dielectrics, the hydroxyl groups easily adsorb water molecules and trap electrons at the semiconductor/dielectric interface, which will reduce the performance and stability of OFETs [35, 36]. Our results also confirm this point. Figures 2 and 4, respectively, show the poor stability of PVA/c-PVA and c-PVP dielectrics and the corresponding C8-BTBT organic transistors. For PVA and c-PVA dielectric transistors, the fluctuant electrical signals in transfer curves of Figure 4(a) can be clearly observed at high humidity. When the humidity increases to 40%, the PVA dielectric transistor cannot be operated, and the c-PVA and c-PVP dielectric transistors also show the obvious performance deterioration with the rapidly increased off-state current. In contrast, the PEI-EP dielectric device can remain with the stable field-effect characteristic. The transfer curves only show slightly changed from 20 to 100% humidity environment. These results indicate that the PEI-EP dielectric device could operate well in high-humidity environment. As far as we know, it is the first paper based on polymer dielectrics that can work in 100% humidity environment [28, 37]. In addition, the high stability of PEI-EP dielectric is favorable for the operation stability of OFETs.

Figure 4(b) shows the cycle stability of the transistors with the continuous switch between on and off states for 1000 s at 20% humidity, and Figure 4(c) records the mobility change in ambient environment for 60 days. For PVA/c-PVA and c-PVP dielectric transistors, the off-state current is increased dramatically, and the mobility decreases sharply, respectively, with 99%, 70%, and 77% degradation in 2 months (Figures 4(b) and 4(c)). In contrast, the PEI-EP dielectric transistor exhibits excellent operating cycle stability under the continuous on and off cycle tests and good environmental stability with a slight decrease of the device mobility (only 22% degradation) after exposure in air ambient for 2 months. These results show that the PEI-EP dielectric brings far better stability of transistors compared with the PVA/c-PVA and c-PVP dielectrics.

### 2.5. Flexibility of PEI-EP Dielectric OFETs

**Figure 5:**
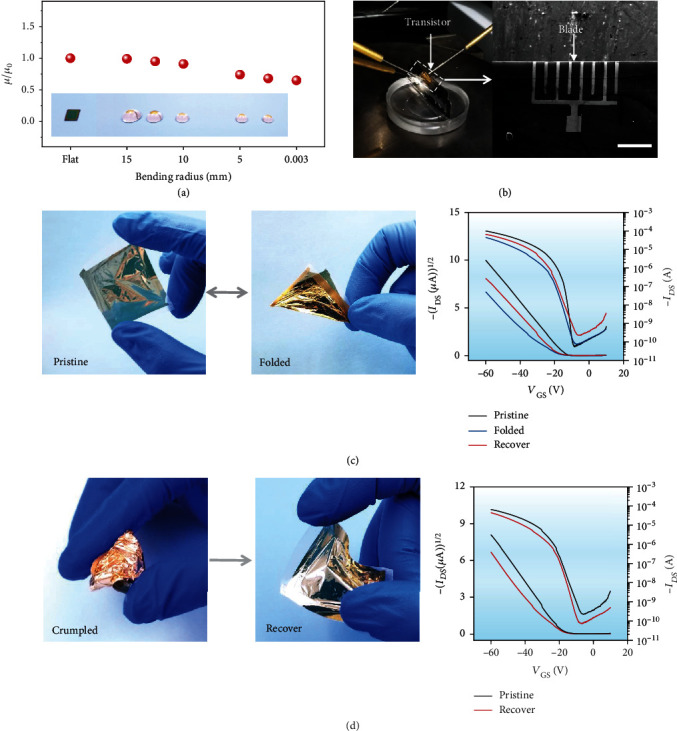
Ultraflexible organic transistors based on PEI-EP dielectric. (a) Normalized mobility at the bending radius from 15 to 0.003 mm. Inset of (a) is the photographs of the OFET conformed onto the glass hemisphere with different bending radius. (b) Photograph of the OFET conformed onto the edge of the blade (bending radius at 0.003 mm) and magnified SEM image (scale bar: 200 *μ*m). (c) Photographs and transfer curves of the device through the folding process to recovery and crumpling to recovery, respectively.

Flexibility and foldability are important characteristics in flexible electronics, which are favorable for the applications of e-skin and e-paper. Here, the transistor flexibility is determined mainly by the dielectric thickness. To further investigate the mechanical flexibility and conformable and foldable capability of the PEI-EP dielectric solution-processed OFET, we transferred the devices onto the 3D spherical surfaces with different bending radius from 15 to 2.5 mm. In addition, the transistor was also adhered to a thin blade with the bending radius down to 0.003 mm.  Figure 5(a) illustrates the normalized *μ*/*μ*_0_ for different bending radius from 15 to 0.003 mm.  Figure 5(b) clearly shows the testing photo and the SEM image of the device at the blade edge. The device presents the normal operation with the weak mobility changes at different bending radius. At the bending radius of 0.003 mm, the mobility is ~70% of the initial mobility. The extremely small bending radius down to 0.003 mm in our organic transistor shows the ultraflexibility of the device, which benefits from the ultraflexible PEI-EP dielectric [38–42]. In addition, as shown in Figure 5(c), the flexible PEI-EP dielectric transistor can be folded, crumpled, and recovered; the excellent flexibility of the PEI-EP dielectric transistor ensures the normal operation of the device with weak performance change (lower than 25%) due to low Young's modulus of PEI-EP as shown in Figure S12.

### 2.6. Universality of PEI-EP as Dielectric for Organic Electronics

**Figure 6:**
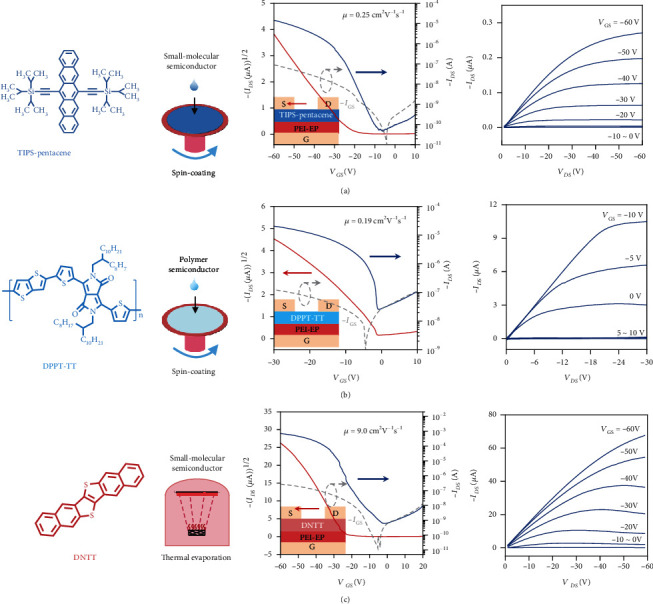
The universality of PEI-EP as dielectric for organic electronics. (a) Spin-coated small-molecular semiconductor TIPS-pentacane. (b) Spin-coated polymer semiconductor DPPT-TT. (c) Thermal evaporation small-molecular semiconductor DNTT. The mobilities are extracted with the capacitance at 1 Hz, and the dotted line in transfer curves is the gate leakage current.

To demonstrate the universality of the PEI-EP as dielectric and its applicability for organic electronics, different organic semiconductors, including TIPS-pentacene, poly[[2,5-bis(2-octyldodecyl)-2,3,5,6-tetrahydro-3,6-dioxopyrrolo [3,4-c]pyrrole-1,4-diyl]-alt-[[2,2′-(2,5-thiophene)bis-thieno(3,2-b) thiophene]-5,5′-diyl]] (DPPT-TT), as well as DNTT, have been used as the active materials for the fabrication of the PEI-EP dielectric OFETs. The chemical structures of TIPS-pentacene, DPPT-TT, and DNTT are, respectively, listed in Figure 6. These semiconductor materials could be successfully deposited on the PEI-EP dielectric forming the uniform thin film, either by solution method or by vapor method. When PEI-EP is used as dielectric, the typical transfer and output characteristics can be obtained for all these organic semiconductor materials including small molecule and polymer and solution-processed and vacuum-deposited organic semiconductors; the AFM morphology images are shown in Figure S13. In addition, high mobilities can be obtained for different semiconductors on the PEI-EP dielectric. Chen's group [43] developed a simple and facile peeling method with the PMMA resist as a postremedy strategy to remove the redundant and protuberant OTS aggregates or other impurities on the OTS/SiO_2_ surface. After peeling treatment, the unprecedented maximum mobility reaches 8.16 cm^2^ V^−1^ s^−1^. Our polymer dielectric PEI-EP based on DNTT semiconductor without any chemical modification exhibited the ultrahigh mobility as high as 9.0 cm^2^ V^−1^ s^−1^, which is the highest mobility based on the DNTT thin-film organic transistor as shown in Table S1. The high field-effect performance and the good film-forming ability of the PEI-EP dielectric show its big application potential as the dielectric for next-generation flexible organic electronics.

## 3. Conclusion

In conclusion, a novel nonhydroxyl polymer dielectric PEI-EP is synthesized and firstly used as the gate dielectric of OFETs. This dielectric material is available for both solution-processed and vacuum-deposited organic semiconductors. The compared results with the conventional PVA/c-PVA and c-PVP dielectrics show the strong resistance capability of PEI-EP to organic solvent and water, good thermal stability, humidity stability, and frequency stability. Compared with the PVA/c-PVA and c-PVP dielectric transistor, the PEI-EP dielectric device could work well within 2 months in different environments from 20 to 100% humidity, with high operation stability. The PEI-EP dielectric solution-processed C8-BTBT transistor array shows a successful yield ratio of 100%, the highest mobility up to 7.98 cm^2^ V^−1^ s^−1^, the average mobility as high as 5.3 cm^2^ V^−1^ s^−1^, and conformable and foldable capability with the bending radius down to 0.003 mm, showing the promising potential of PEI-EP as dielectric of solution-processed flexible organic electronics. In addition, with PEI-EP dielectric, both solution-processed and vacuum-deposited transistors present high field-effect performance, suggesting that PEI-EP is a promising gate dielectric candidate for OFETs. These results demonstrate the outstanding advantages of PEI-EP as the dielectric material of OFETs and provide a solution for the challenging problem in fabrication of solution-processed flexible OFETs.

## 4. Materials and Methods

### 4.1. Materials

Polyethyleneimine (PEI) (98%, Mw = 10000 g mol^−1^), epoxy resins (EP) (98%, Mw = 500 g mol^−1^), PVA (Mw = 205000 g mol^−1^), PVP (Mw = 205000 g mol^−1^), poly(melamine-co-formaldehyde) (PMF), glutaraldehyde (GA), C8-BTBT (>99%), TIPS-pentacene (>99%), DPPT-TT (99%), and DNTT (99%) were purchased from Sigma-Aldrich. OTS (95%) was purchased from Acros. Chloroform, chlorobenzene, heptane, isopropanol, acetone, and propylene glycol monomethyl ether acetate were obtained from Beijing Chemical Reagent Co., Ltd. without further purification.

### 4.2. Preparation of the Polymer Dielectric PEI-EP

0.25 g PEI and 1 g EP were dissolved in 17 mL chloroform with a solution concentration of 7 wt%. Then, the mixed solution of PEI and EP was stirred overnight to form uniform solution. The solution was spin-coated through a PVDF filter with a diameter of 0.2 *μ*m on the cleaned Si wafer. After that, the PEI-EP thin film was placed in the 70°C oven for 2 h to promote the crosslinking reaction. The thickness of the PEI-EP dielectric is 633 nm as shown in Figure S14.

### 4.3. Substrate Preparation and Fabrication of Flexible PEI-EP Dielectric OFET Array

The Si substrates were cleaned by sonication in acetone and isopropanol for 10 min and subsequently dried by nitrogen. Then, the OTS treatment was proceeded by dipping the Si wafers into OTS solution (OTS: heptane = 1 : 1000 by volume). Next, 50 nm Au was deposited on the OTS-treated Si wafer by vacuum (0.1 Å s^−1^) as the bottom electrode. The mixed solution of PEI and EP was spin-coated on the Au at a spinning rate of 6000 rpm and thermal annealing at 70°C for 2 h. C8-BTBT was dissolved in chlorobenzene (3-10 mg/mL) at different spinning speeds from 1000 to 8000 rpm for 30 s. Finally, 30 nm Au source and drain electrodes were thermally deposited by vacuum deposition (0.1 Å s^−1^). The whole flexible polymer dielectric PEI-EP-based organic transistor was easily peeled off from Si wafer.

### 4.4. Fabrication of Comparable PVA/c-PVA and c-PVP Dielectric OFET

The bottom-gated top contact device was prepared on the cleaned Si wafer. 7 wt% concentration PVA/c-PVA (PVA : GA = 10 : 1) and c-PVP (PVP : PMF = 10 : 1) solution were spin-coated on Si wafer. Next, PVA/c-PVA and c-PVP films were annealed at 100°C for 2 h. The thickness of PVA/c-PVA and c-PVP dielectric is 527 nm/605 nm and 468 nm as shown in Figure S13. After that, 5 mg/mL C8-BTBT semiconductor was spin-coated on the PVA/c-PVA and c-PVP thin film. Finally, 30 nm Au source and drain electrodes were deposited by vacuum deposition at a rate of 0.1 Å s^−1^.

### 4.5. Fabrication of TIPS-Pentacene, DPPT-TT, and DNTT-Based Transistor

The cleaned Si wafer was used as the bottom gate electrode. Next, the mixed solution of PEI and EP was spin-coated on the Si wafer and thermally annealed at 70°C for 2 h. 3 mg/mL TIPS-pentacene and DPPT-TT were spin-coated on PEI-EP thin film at the spinning speed of 2000 rpm. After that, the DPPT-TT was annealed at 150°C for 30 min. DNTT thin film was deposited by thermal evaporation at a rate of 0.15 Å s^−1^ at a substrate temperature of 60°C. Finally, 30 nm Au was deposited as source and drain electrodes, respectively.

### 4.6. Characterization

Optical microscopy investigations were performed with an Olympus BX51 microscope and a Keyence VHX-5000 (Keyence, Japan). AFM measurements were performed in air with a Bruker Dimension Icon instrument (Bruker, Berlin, Germany). SEM images were obtained by the Philips XL30 instrument. FTIR spectra were obtained by the Nicolet iS10 (Thermo Scientific). The capacitance of PVA/c-PVA, c-PVP, and PEI-EP was measured by the IM3590 chemical impedance analyzer (Hioki Electric Co., Ltd) from 200 kHz to 0.1 Hz.

The electrical characteristics of OFET devices were recorded with a Keithley 4200 SCS and a Cascade M150 probe station at room temperature in air. All the field-effect parameters are calculated with the standard equation in the saturation regime. The standard equation is

(1) $$\begin{array}{c} I_{DS}=\frac{\mu {WC}_{i}}{2L}{\left(V_{G}-V_{T}\right)}^{2}, \end{array}$$

where *W* and *L* are the channel width and channel length, respectively, *C*_*i*_ is the capacitance per unit area of PEI-EP which is 2.3 nF/cm^2^ at 1 Hz, *I*_*DS*_ is the drain current, and *V*_*GS*_ is the gate voltage.

## Data Availability

All data needed to evaluate the conclusions in the paper are presented in the paper and/or Supplementary Materials. Additional data related to this paper may be requested from the authors.

## Supplementary Materials

Supplementary Materials Figure S1: FTIR spectra of PEI-EP. Figure S2: transmittance of PEI-EP at visible light. Figure S3: contact angle of H_2_O on the PEI-EP surface. Figure S4: FTIR spectra and swelling ratio of PEI-EP immersed in H_2_O, chloroform, hexane, and acetone. Figure S5: the AFM image of the PEI-EP dielectric. Figure S6: AFM image of Au/C8-BTBT/PEI-EP/Au structure with a thickness at 890 nm. Figure S7: the mass of OFET is only 0.2 mg/cm^2^. Figure S8: AFM morphology images of the spin-coated C8-BTBT films at different concentrations. Figure S9: AFM morphology images of the spin-coated C8-BTBT films at spinning speeds from 1000 to 8000 rpm. Figure S10: output curve of the PEI-EP dielectric OFET. Figure S11: double sweep transfer and gate leakage current of the PEI-EP dielectric OFET. Figure S12: Young's modulus AFM image of the PVA, c-PVA, c-PVP, and PEI-EP dielectric. Figure S13: the AFM image of solution-processed TIPS-pentacene on the PEI-EP dielectric. Table S1: the electrical performance comparable to the DNTT organic transistor. Figure S14: the thickness of PVA, c-PVA, c-PVP, and PEI-EP by AFM image.
