# Supplementary material for: Highly Stable Nonhydroxyl Antisolvent Polymer Dielectric: A New Strategy towards High-Performance Low-Temperature Solution-Processed Ultraflexible Organic Transistors for Skin-Inspired Electronics
Source: Research (Wash D C). 2021 Dec 8;2021:9897353. doi: 10.34133/2021/9897353 (PMC8678616; doi:10.34133/2021/9897353)
Supplement: Supplementary Materials — Figure S1: FTIR spectra of PEI-EP. Figure S2: transmittance of PEI-EP at visible light. Figure S3: contact angle of H2O on the PEI-EP surface. Figure S4: FTIR spectra and swelling ratio of PEI-EP immersed in H2O, chloroform, hexane, and acetone. Figure S5: the AFM image of the PEI-EP dielectric. Figure S6: AFM image of Au/C8-BTBT/PEI-EP/Au structure with a thickness at 890 nm. Figure S7: the mass of OFET is only 0.2 mg/cm2. Figure S8: AFM morphology images of the spin-coated C8-BTBT films at different concentrations. Figure S9: AFM morphology images of the spin-coated C8-BTBT films at spinning speeds from 1000 to 8000 rpm. Figure S10: output curve of the PEI-EP dielectric OFET. Figure S11: double sweep transfer and gate leakage current of the PEI-EP dielectric OFET. Figure S12: Young's modulus AFM image of the PVA, c-PVA, c-PVP, and PEI-EP dielectric. Figure S13: the AFM image of solution-processed TIPS-pentacene on the PEI-EP dielectric. Table S1: the electrical performance comparable to the DNTT organic transistor. Figure S14: the thickness of PVA, c-PVA, c-PVP, and PEI-EP by AFM image. [file 9897353.f1.docx]

**Supplementary Materials**

A Highly Stable Nonhydroxyl Antisolvent Polymer Dielectric for Low-Temperature Solution-Processed Ultraflexible Organic Transistors

**Authors**

Mingxin Zhang^1^, Cong Zhang^1^, Yahan Yang^1^, Hang Ren^1^, Junmo Zhang^1^, Xiaoli Zhao^1^, Yanhong Tong^1^, Qingxin Tang^1^*, and Yichun Liu^1^*

**Affiliations**

^1^ Centre for Advanced Optoelectronic Functional Materials Research and Key Laboratory of UV-Emitting Materials and Technology, Ministry of Education, Northeast Normal University, Changchun 130024, P. R. China.

*Correspondence should be addressed to Qingxin Tang; [tangqx@nenu.edu.cn](mailto:tangqx@nenu.edu.cn); Yichun Liu; ycliu@nenu.edu.cn.


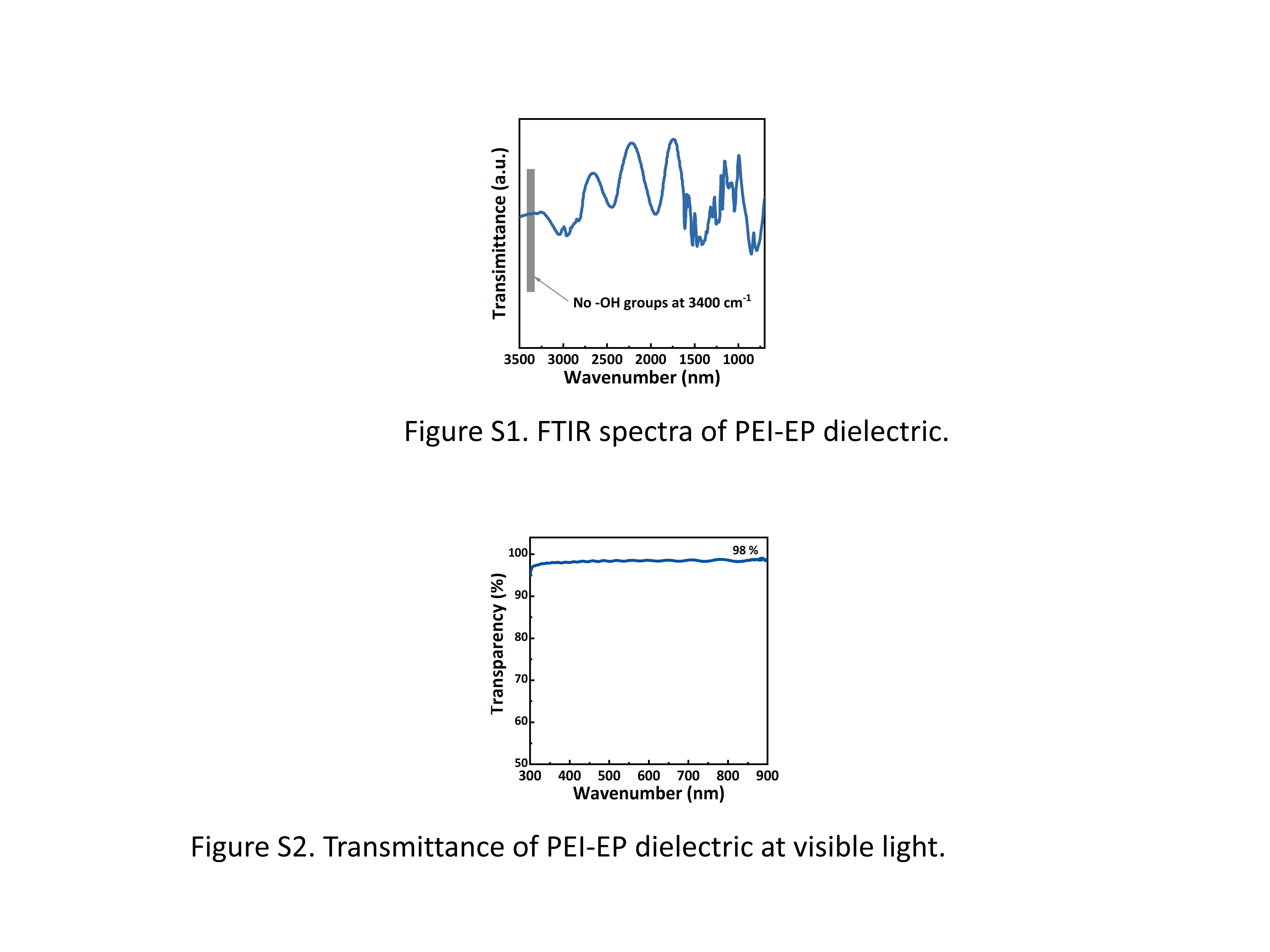


**Figure S1.** FTIR spectra of PEI-EP.


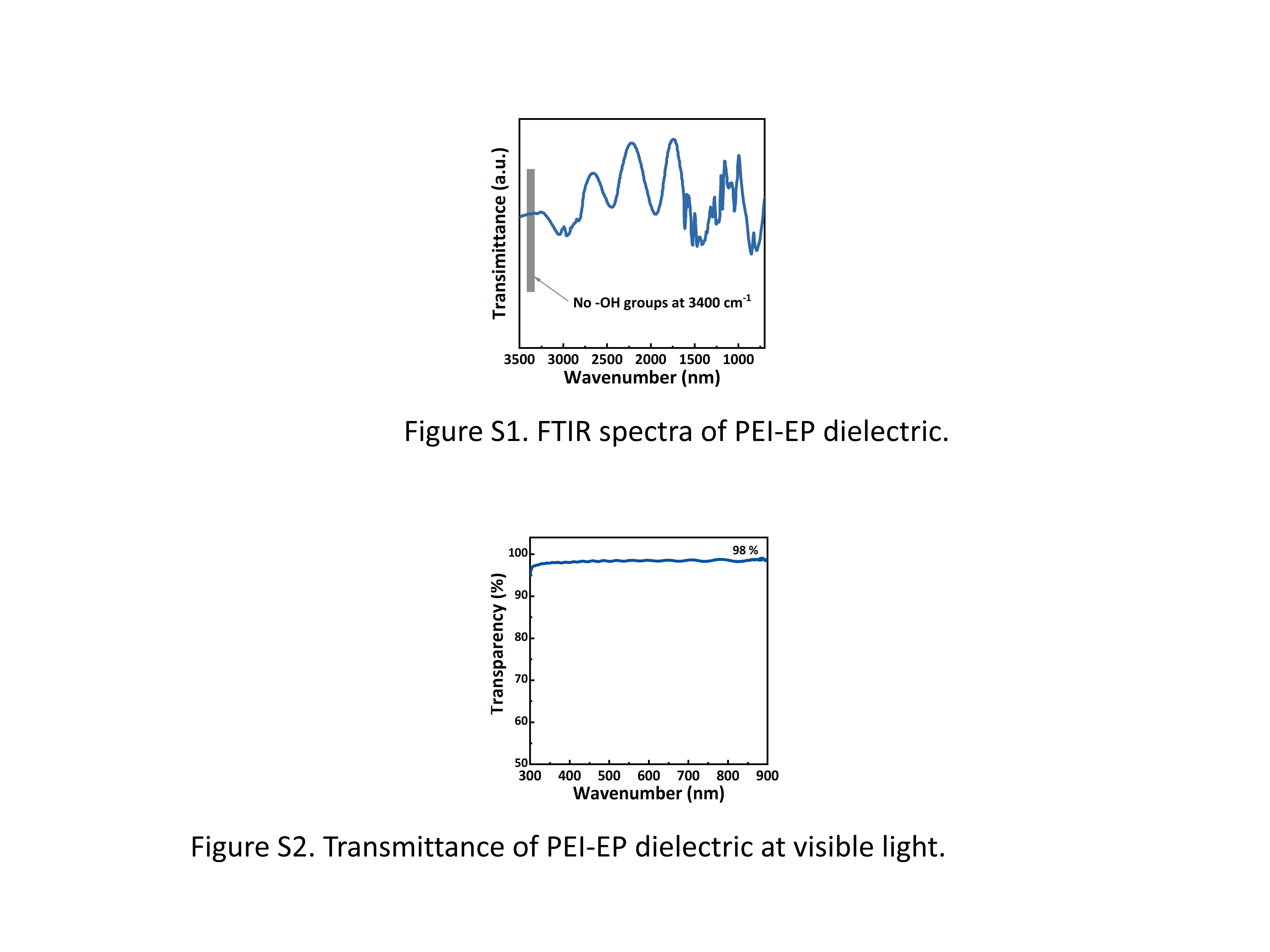


**Figure S2.** Transmittance of PEI-EP at visible light.


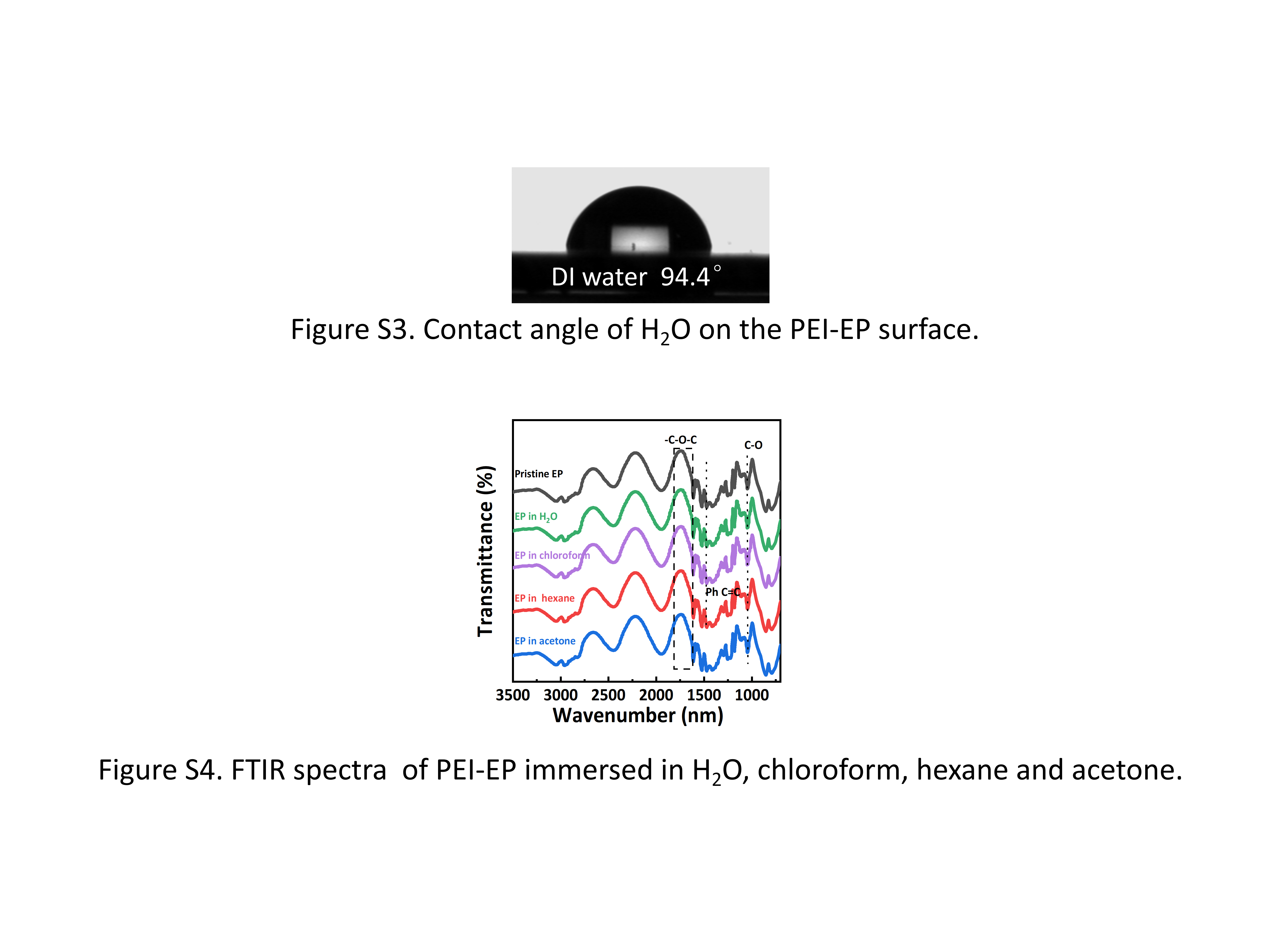


**Figure S3.** Contact angle of H_2_O on the PEI-EP surface.


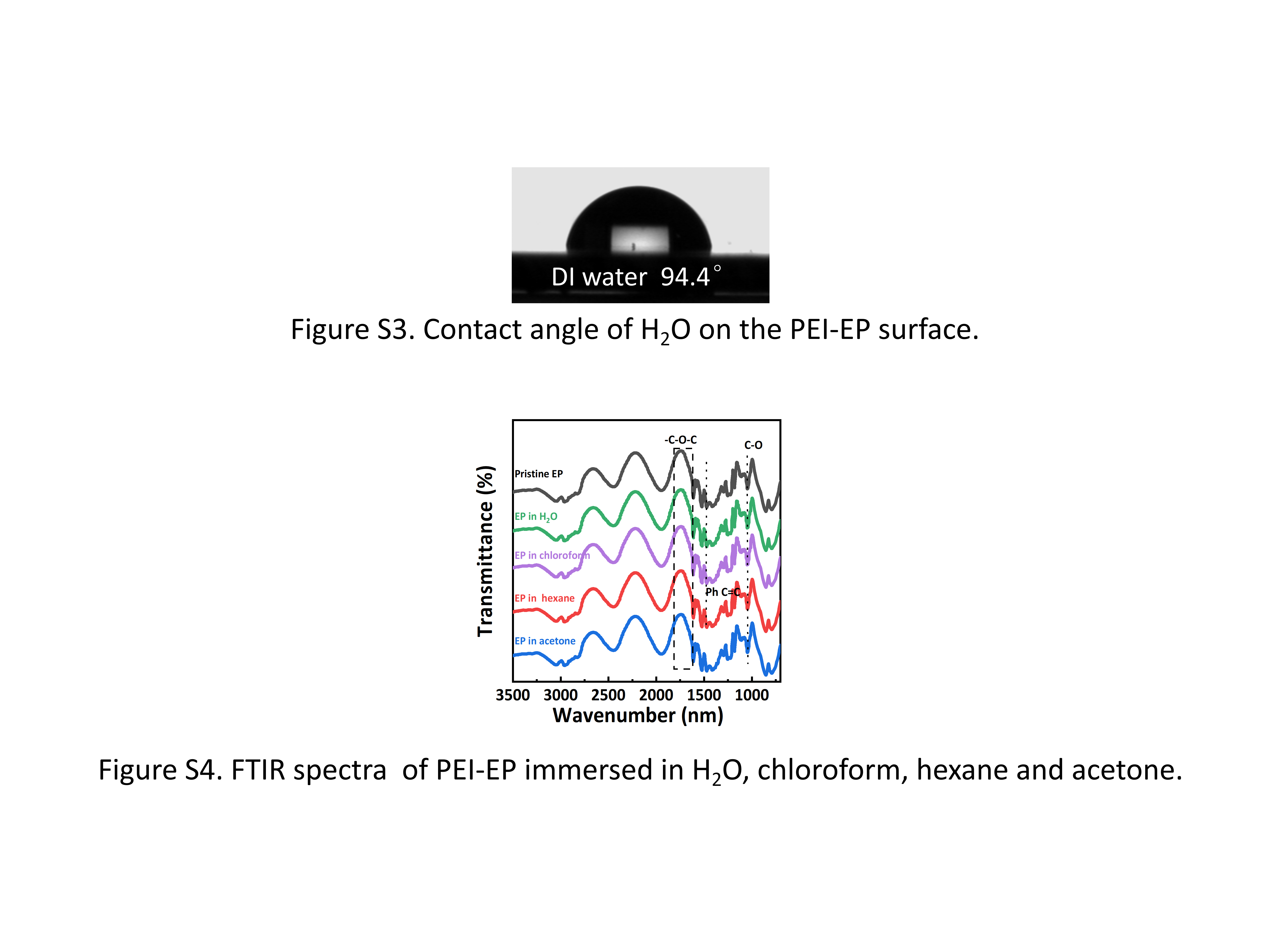

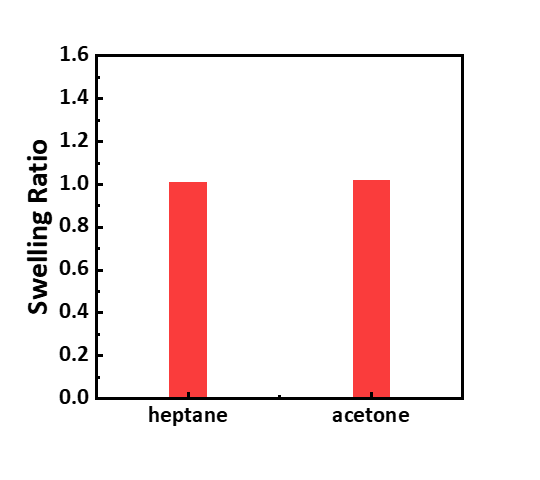


**Figure S4.** FTIR spectra and swelling ratio of PEI-EP immersed in H_2_O, chloroform, hexane and acetone.

**
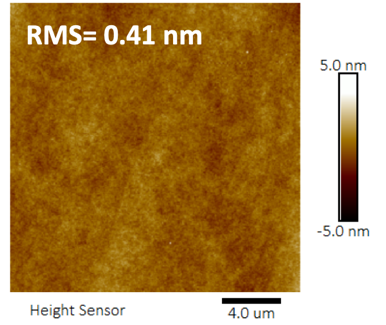
**

**Figure S5. The AFM image of PEI-EP dielectric.**


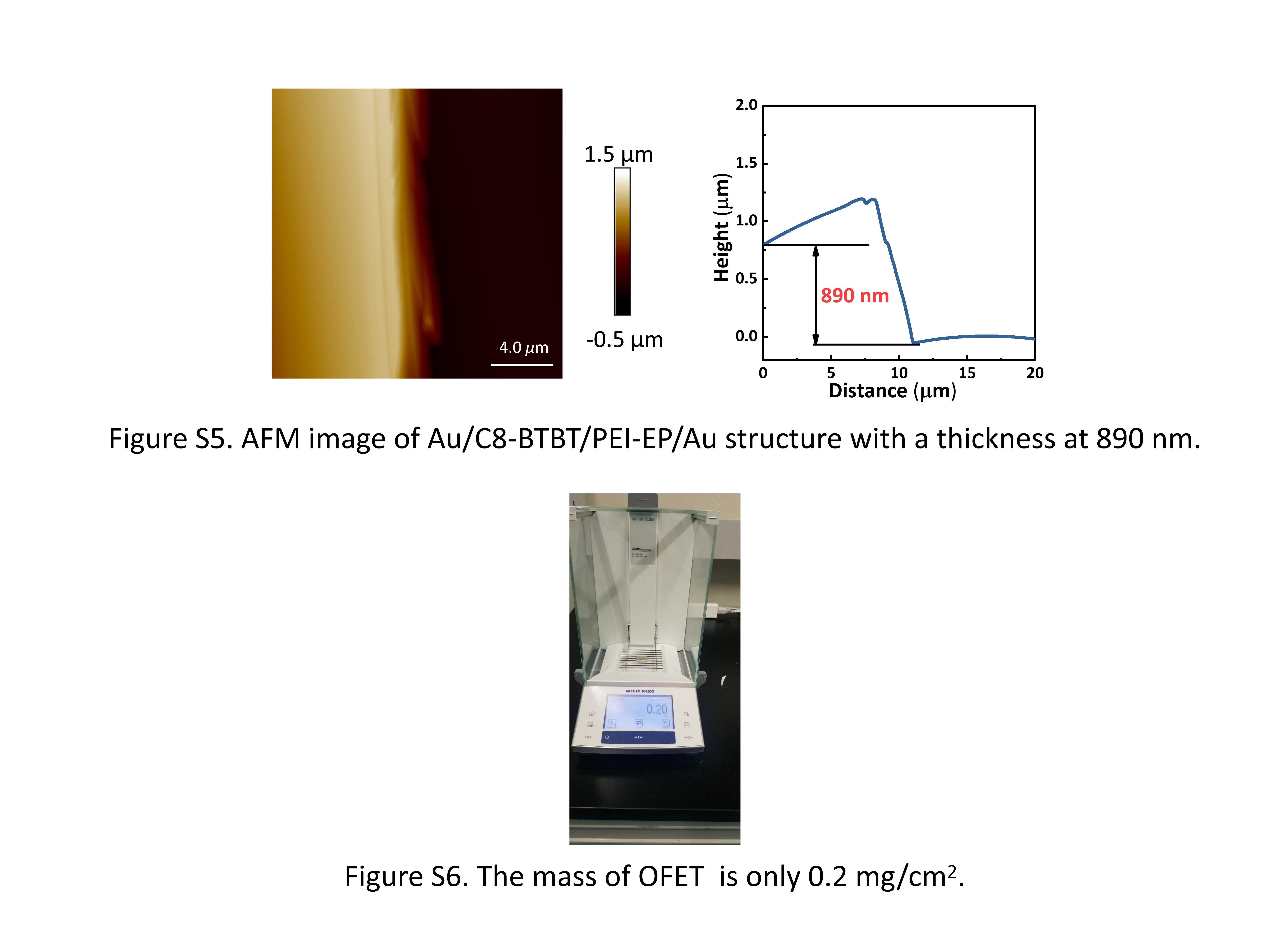


**Figure S6.** AFM image of Au/C8-BTBT/PEI-EP/Au structure with a thickness at 890 nm.


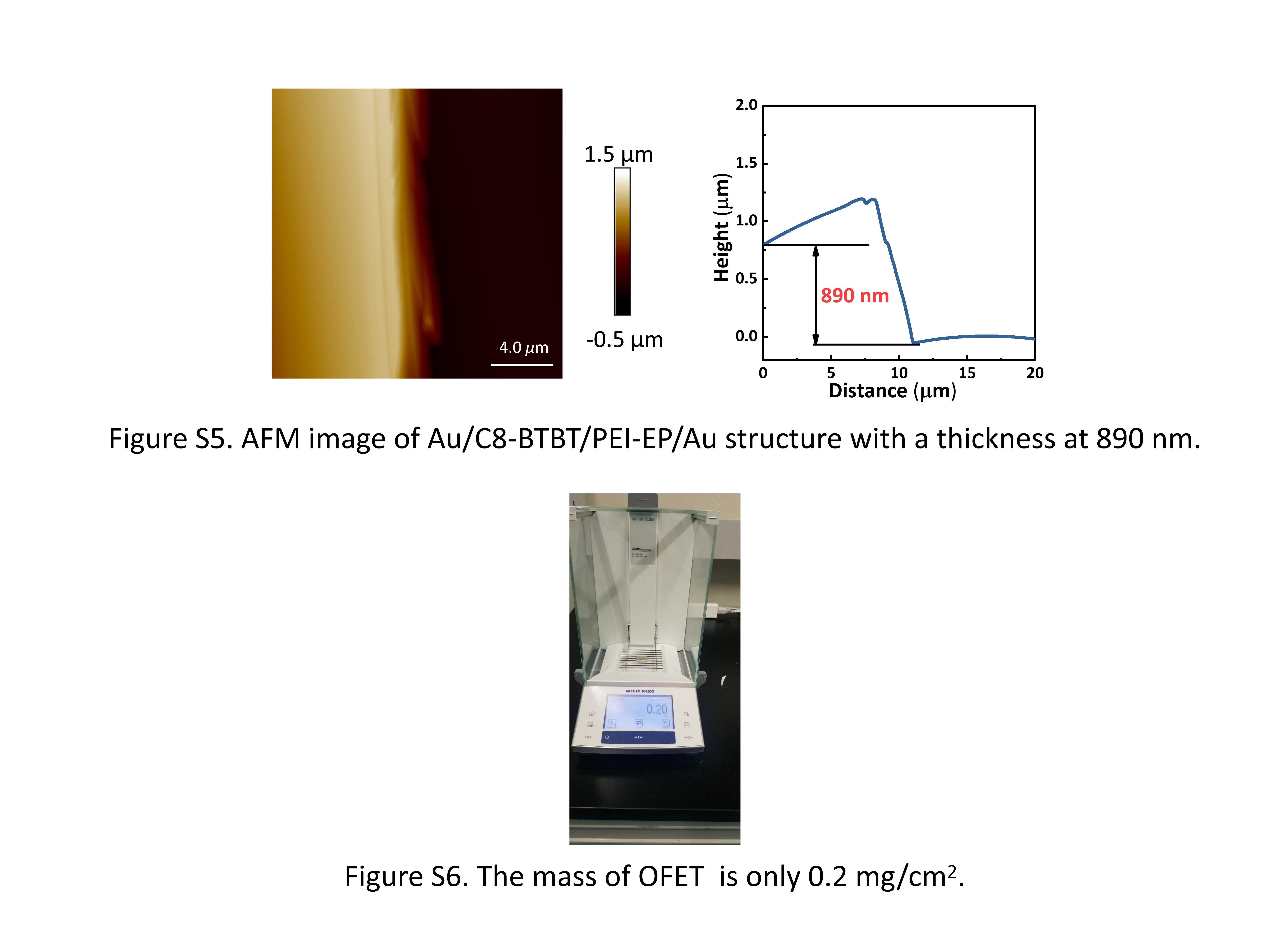


**Figure S7.** The mass of OFET is only 0.2 mg/cm^2^.


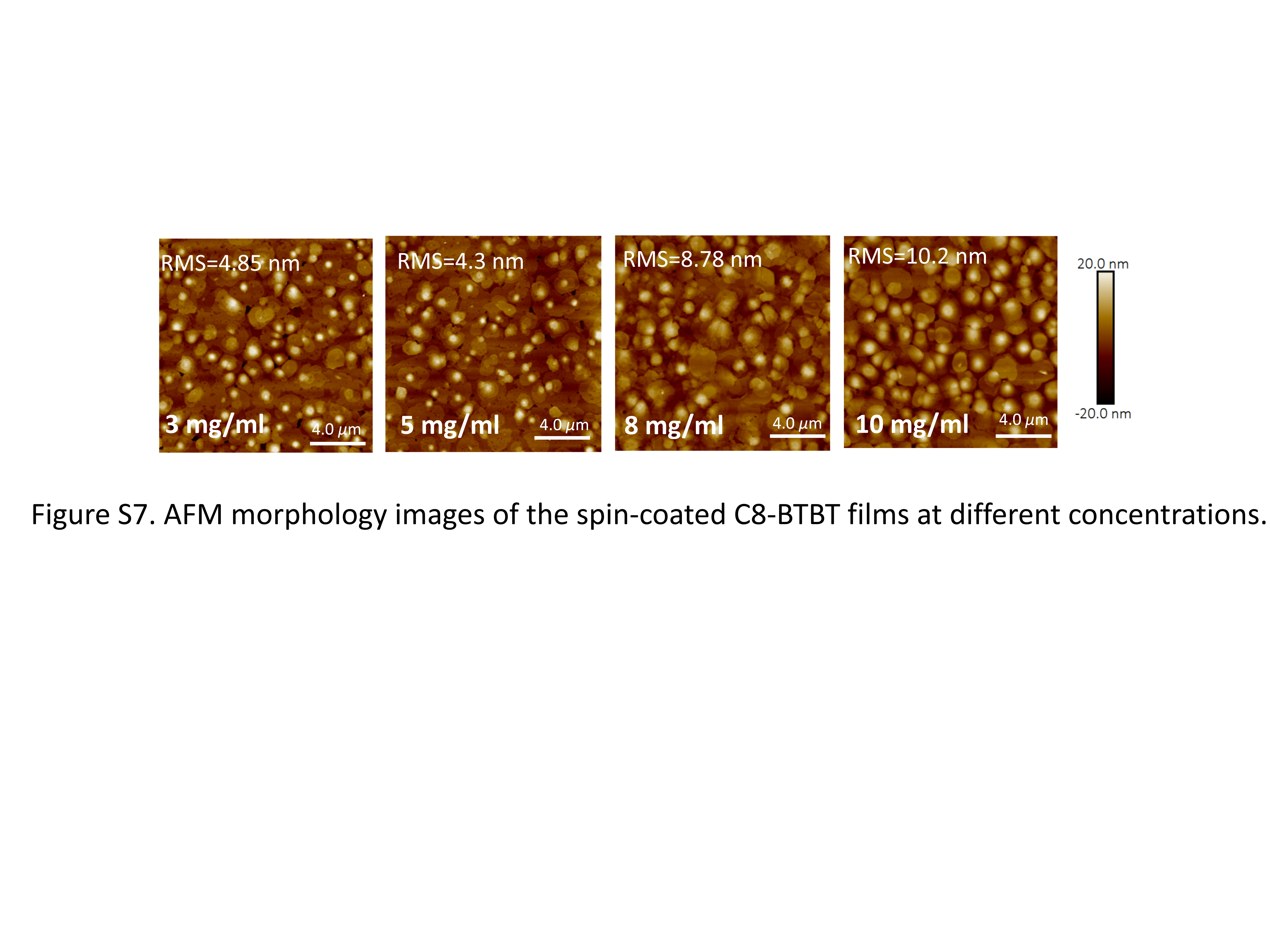


**Figure S8.** AFM morphology images of the spin-coated C8-BTBT films at different concentrations.


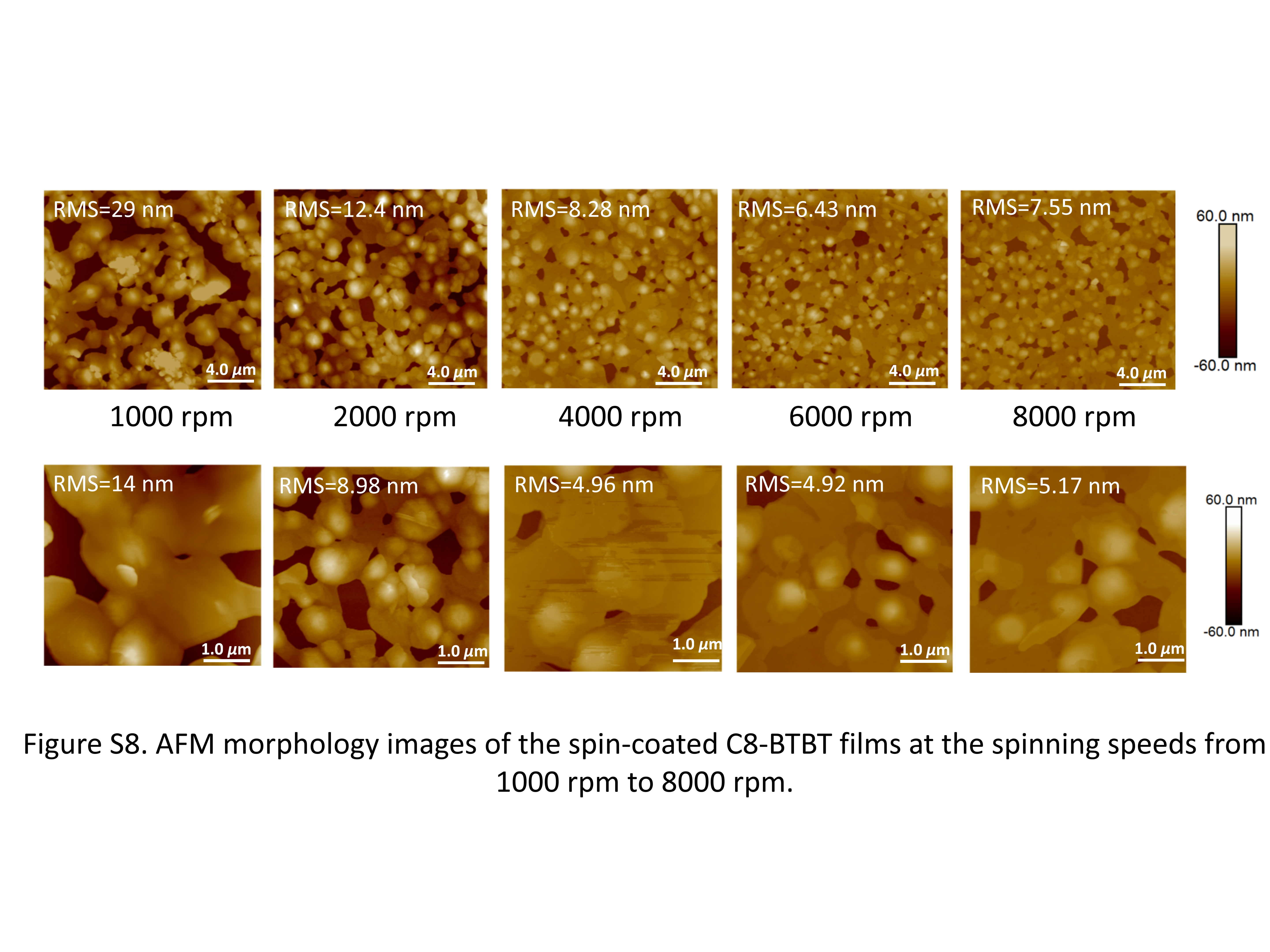


**Figure S9.** AFM morphology images of the spin-coated C8-BTBT films at the spinning speeds from 1000 to 8000 rpm.


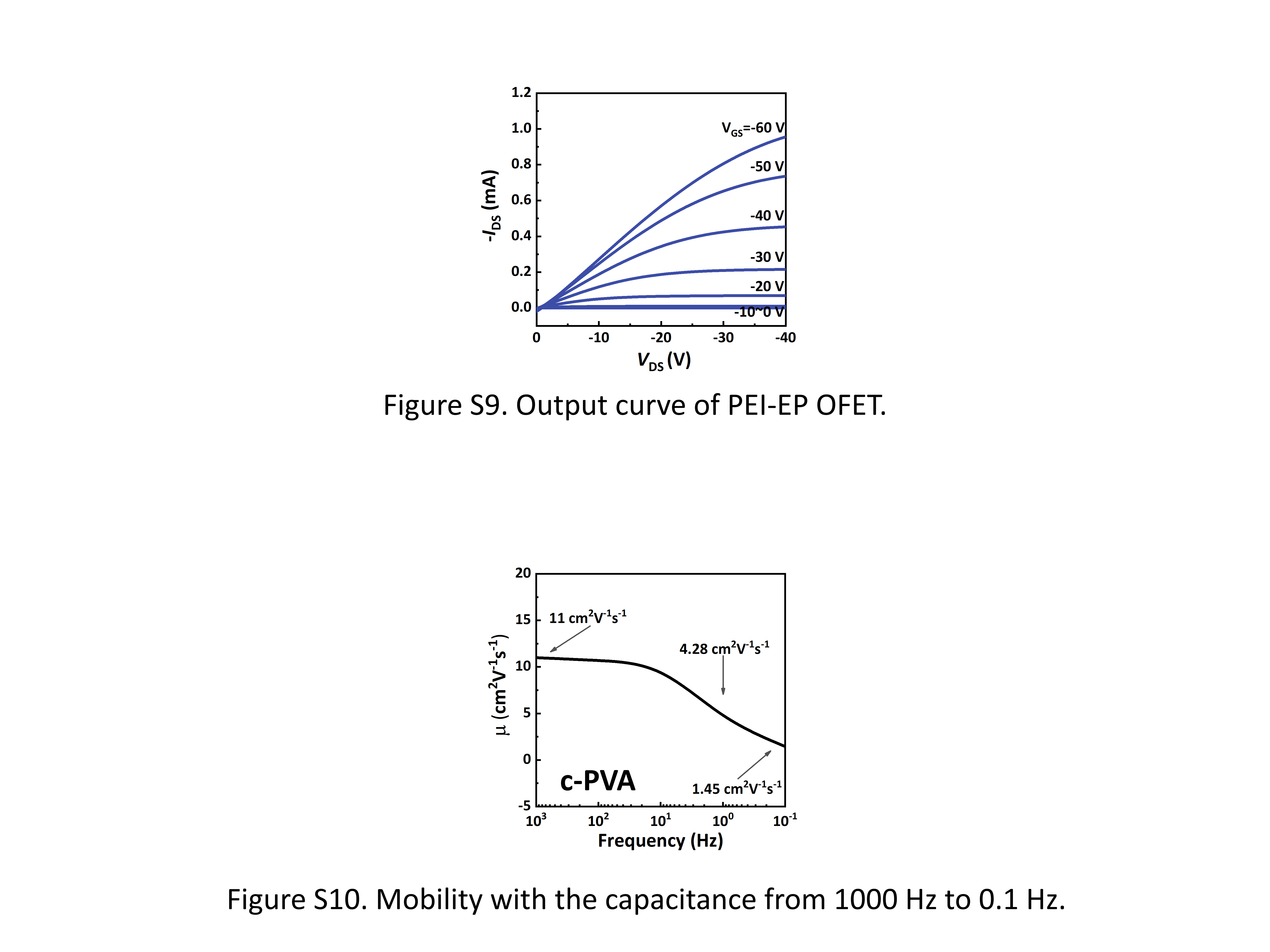


**Figure S10.** Output curve of the PEI-EP dielectric OFET.


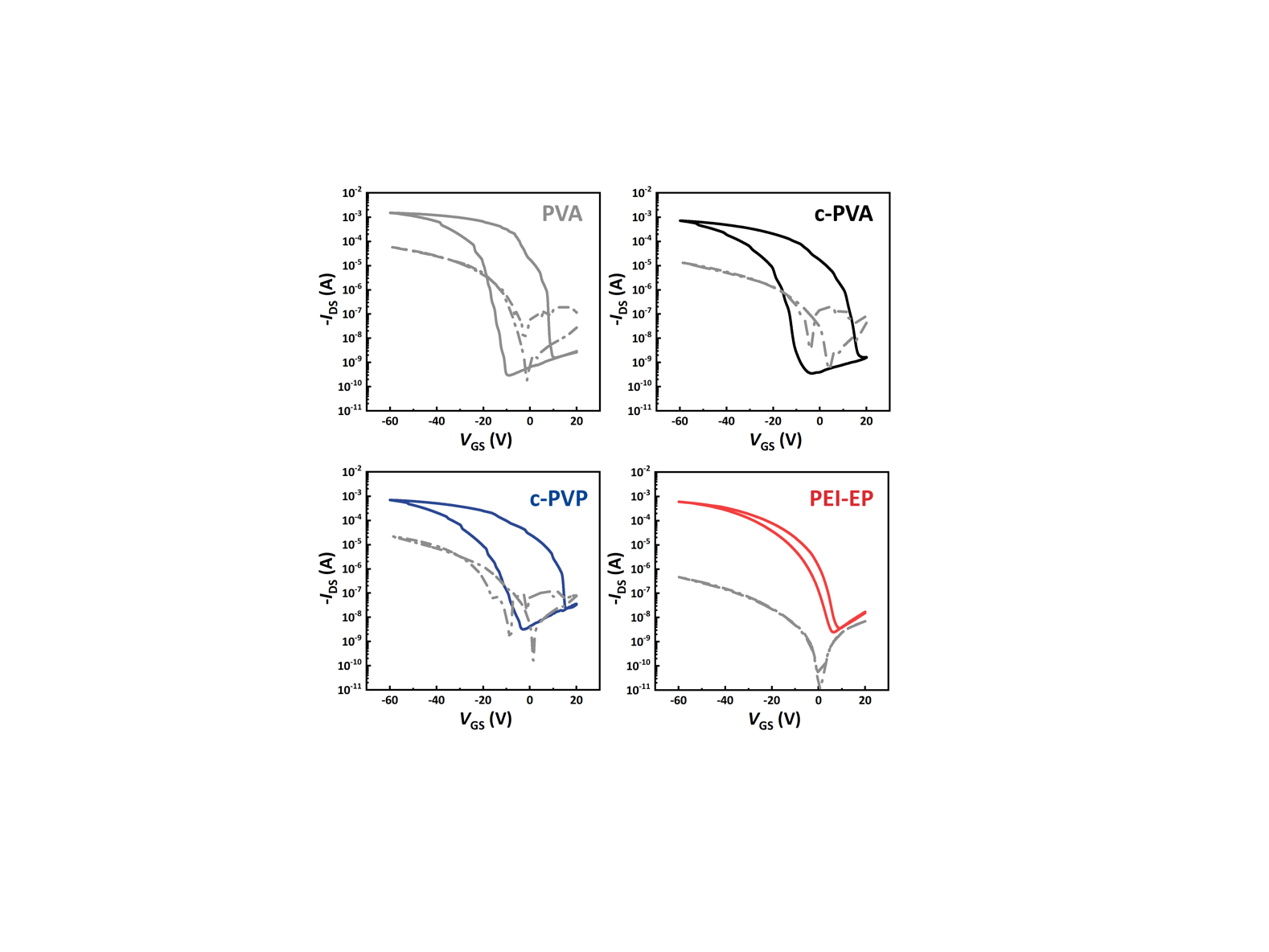


**Figure S11.** Double sweep transfer and gate leakage current of the PEI-EP dielectric OFET.


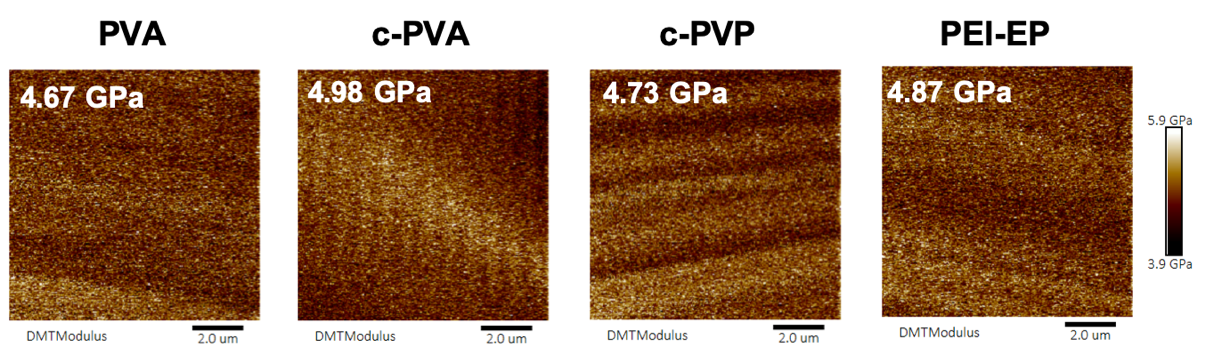


**Figure S12.** The Young's modulus AFM image of the PVA, c-PVA, c-PVP and PEI-EP dielectric.


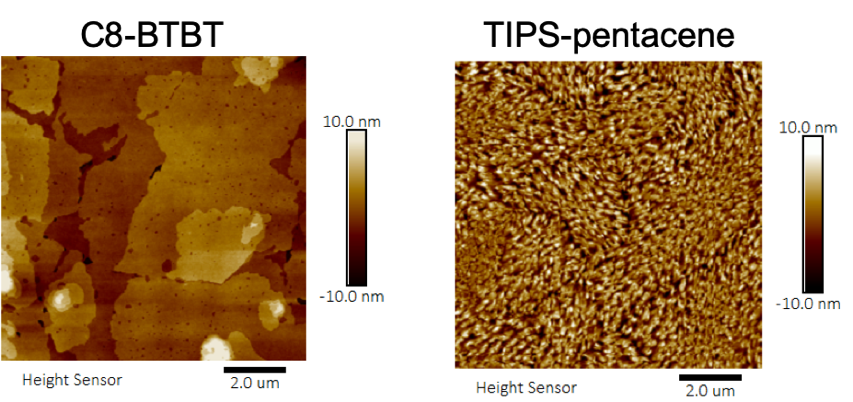


**Figure S13.** The AFM image of solution-processed TIPS-pentacene on the PEI-EP dielectric.

**Table S1.** The electrical performance comparable of DNTT organic transistor.

|  | **Dielectric** | **OSC** | **Electrode** | **Frequency**  **（Hz）** | **Capacitance**  **（nF/cm^2^）** | **μ**  **(cm^2^/Vs)** | **Ref.** |
| --- | --- | --- | --- | --- | --- | --- | --- |
| 1 | SiO_2_ | DNTT | Au | / | / | 2.8 | [1] |
| 2 | SiO_2_ | DNTT | Au | / | 11.5 | 3.02 | [2] |
| 3 | SiO_2_ | DNTT | Au | 100000 | 2.86 | 3.35 | [3] |
| 4 | SiO_2_/AlO_2_ | DNTT | Au | / | 34 | 4.4 | [4] |
| 5 | SiO_2_ | DNTT | Au | / | 10 | 6.08 | [5] |
| 6 | SiO_2_ | DNTT | Au | / | 34.5 | 8.16 | [6] |
| 7 | AlO_2_ | DNTT | Ag | / | 750 | 0.21 | [7] |
| 8 | AlO_2_ | DNTT | Au | / | 700 | 1.1 | [8] |
| 9 | AlO_2_ | DNTT | Au | / | / | 1.51 | [9] |
| 10 | AlO_2_ | DNTT | Au | / | / | 3.3 | [10] |
| 11 | Inoflex T3 | DNTT | Au | / | / | 0.1 | [11] |
| 12 | PEMA/PMAO | DNTT | Au | 60 | 120 | 0.11 | [12] |
| 13 | PEMA | DNTT | Au | 40 | 56.7 | 0.17 | [13] |
| 14 | TPGDA | DNTT | Au | / | 13 | 0.4 | [14] |
| 15 | SiO_2_/PMMA | DNTT | Au | / | / | 0.7 | [15] |
| 16 | PS | DNTT | Ag | / | / | 0.95 | [16] |
| 17 | TPGDA | DNTT | Au | / | / | 1.0 | [17] |
| 18 | PMMA | DNTT | Au | / | / | 1.0 | [18] |
| 19 | PS | DNTT | Au | / | 2.37 | 1.0 | [19] |
| 20 | DC-2577 | DNTT | Au | / | 6 | 5.48 | [20] |
| **21** | **PEI-EP** | **DNTT** | **Au** | **1** | **2.3** | **9.0** | **Our work** |


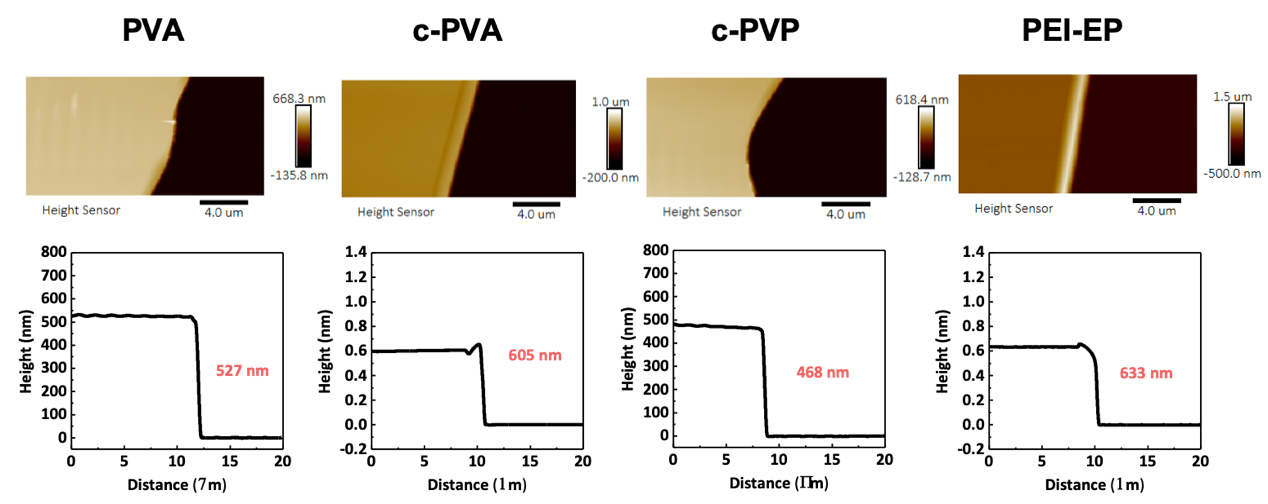


**Figure S14.** The thickness of PVA, c-PVA, c-PVP and PEI-EP by AFM image.
